# Sonority as a Phonological Cue in Early Perception of Written Syllables in French

**DOI:** 10.3389/fpsyg.2020.558443

**Published:** 2020-10-15

**Authors:** Méghane Tossonian, Ludovic Ferrand, Ophélie Lucas, Mickaël Berthon, Norbert Maïonchi-Pino

**Affiliations:** ^1^CNRS, LAPSCO, Université Clermont Auvergne, Clermont-Ferrand, France; ^2^Unité de Recherche en Neurosciences Cognitives (UNESCOG), Université libre de Bruxelles, Brussels, Belgium

**Keywords:** visual word processing, sonority, markedness, syllable, illusory conjunctions, French, phonological universals

## Abstract

Many studies focused on the letter and sound co-occurrences to account for the well-documented syllable-based effects in French in visual (pseudo)word processing. Although these language-specific statistical properties are crucial, recent data suggest that studies that go all-in on phonological and orthographic regularities may be misguided in interpreting how—and why—readers locate syllable boundaries and segment clusters. Indeed, syllable-based effects could depend on more abstract, universal phonological constraints that rule and govern how letter and sound occur and co-occur, and readers could be sensitive to sonority—a universal phonological element—for processing (pseudo)words. Here, we investigate whether French adult skilled readers rely on universal phonological sonority-related markedness continuum across the syllable boundaries for segmentation (e.g., from marked, illegal intervocalic clusters /*zl*/ to unmarked, legal intervocalic clusters /*lz*/). To address this question, we ran two tasks with 128 French adult skilled readers using two versions of the illusory conjunction paradigm (Task 1 without white noise; Task 2 with white noise). Our results show that syllable location and segmentation in reading is early and automatically modulated by phonological sonority-related markedness in the absence or quasi-absence of statistical information and does not require acoustic-phonetic information. We discuss our results toward the overlooked role of phonological universals and the over-trusted role of statistical information during reading processes.

## Introduction

In syllable-timed languages such as French, there is robust evidence that supports the syllable as a perceptual and functional unit that early and automatically mediates the access to the lexicon and drives the segmentation strategies in the first steps of visual (pseudo)word processing in adults (e.g., Ferrand et al., 1996; Mathey and Zagar, 2002; Doignon and Zagar, 2005; Mathey et al., 2013). Uncontroversial literature—including electrophysiological markers—either in tasks that do or do not tap into lexical access apparently supports the leading role of sound and letter co-occurrences within and across syllables to account for these syllable effects (e.g., initial syllable frequency, etc.; e.g., Colé et al., 1999; Goslin et al., 2006; Chetail and Mathey, 2009a,b; Doignon-Camus et al., 2009a; Maïonchi-Pino et al., 2010; Chetail et al., 2012; Mahé et al., 2014; Chetail, 2015).

This *a priori* focus on an “all is a matter of statistical properties” could not be the sole and unique approach to account for these syllable-based effects. Questions come from controversies—and inconsistencies—in studies that banked on quantifiable orthographic statistical—and distributional—properties, like the *bigram trough* (Seidenberg, 1987), either in children (e.g., in French, Kandel et al., 2011; Doignon-Camus et al., 2013; Doignon-Camus and Zagar, 2014; Maïonchi-Pino et al., 2015a) or in adults (e.g., in French, Doignon and Zagar, 2005, 2006; Doignon-Camus et al., 2009b; in Spanish, Carreiras et al., 1993; Conrad et al., 2009; in English, Rapp, 1992; Muncer et al., 2014). The clincher of the bigram trough hypothesis to account for mapping letter clusters that frequently co-occur onto syllables and define perceptual syllable boundaries (i.e., “AN.VIL”; e.g., Seidenberg, 1987; Doignon-Camus et al., 2009a,b, 2013) is that letter co-occurrences straddling syllable boundaries (e.g., the bigram “NV” in “ANVIL”) are of lower frequency than letter co-occurrences preceding or after syllable boundaries (i.e., “AN” and “VI”). However, these language-specific properties could be insufficient or at least over-trusted to account for syllable-based effects. Indeed, although the bigram trough—as well as the initial syllable frequency, etc.—reflects orthographic regularities through the distribution of letters within words (e.g., Chetail, 2015), more knowledge is required about how and how frequently letter (co-)occurrences in specific positions in a given language are phonologically guided? Of interest is what appears to be a statistically grounded cue that actually conforms to general phonological restrictions that allow or ban some associations and, therefore, define orthographic—and phonotactic—regularities, which may vary from a language to another (e.g., /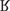
b/, “RB” never occurs in French onset clusters but does occur in intervocalic positions).

For instance, within the framework of the generative *Optimality Theory* (e.g., Prince and Smolensky, 2004), *universal phonological grammar* rules and governs how—and how often—phonemes occur and co-occur within specific positions through a system of hierarchically ranked *violable* phonological constraints. These constraints do not differ from language to language. What differentiates languages relies on how a language orders these constraints and minimally transgress—or maximally respect—them.

One of the phonological universals—here *sonority*, which underlies the markedness constraints—has been demonstrated to crucially contribute to syllable-based effects in visual (pseudo)word processing in adults, children who are learning to read, and children with developmental dyslexia in French (e.g., Bedoin and Dissard, 2002; Marouby-Terriou and Denhière, 2002; Fabre and Bedoin, 2003; Maïonchi-Pino et al., 2012a,b, 2015a; Maïonchi-Pino et al., 2020).

Sonority is described as a universal phonological element that categorizes all speech sounds into a hierarchical acoustic–phonetic scale and drives the organization within and across syllables (e.g., Barlow, 2016; Parker, 2017). Parker (2002, 2008) proposes that sonority is a phonological property of sounds with concrete, quantifiable physical and perceptual parameters, the acoustic intensity being the most reliable correlate (see Supplementary Figure 1). Based on individual sonorities, some sonority-based linguistic principles have been developed to describe what optimal syllables and syllable contacts should be (e.g., the *Sonority Sequencing Principle*, e.g., Clements, 1990, 2006; the *Syllable Contact Law*, e.g., Murray and Vennemann, 1983; Vennemann, 1988). Although an optimal syllable should be an onset that tends to grow toward a maximum sonority at the vowel and fall to a minimum sonority at the coda (e.g., “carbon”), an optimal syllable contact should prohibit a sonority rise to promote a steep fall across syllable boundaries (e.g., “car.bon” > “ma.trix”; the dot stands for the expected location of the syllable boundary).^1^

In three studies conducted in French, Doignon and Zagar (2005, 2006) and Doignon-Camus et al. (2013) found that neither phonological properties (i.e., initial CV/CVC syllable frequency) nor orthographic properties (i.e., frequency of the initial bigram/trigram or the frequency of the bigram that precedes, straddles, or follows the syllable boundaries) were essential. Rather, a sonority-based organization optimizes syllable location and segmentation of intervocalic C_1_C_2_ clusters. This is the case with “sonorant coda–obstruent onset” sonority profiles (SPs henceforth; e.g., “LT” in “VULTI” or “LP” in “TOLPUDE”). Such SPs facilitate the segmentation of pseudowords like “VUL.TI” and “TOL.PUDE” compared with “TL” in “DATLORE” (e.g., Bedoin and Dissard, 2002; Fabre and Bedoin, 2003; Maïonchi-Pino et al., 2012a,b, 2015a). Despite attractive conclusions, one potential limitation of these studies is that their observations ensue from some attested C_1_C_2_ with quantifiable orthographic and phonological statistical properties (e.g., /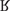
b/, “RB”). There was no clear dissociation between attested (that do exist) vs. unattested clusters (that do not exist) either in onset or intervocalic position for the French language, whereas adults and children could have inferred the well-formedness of a C_1_C_2_ cluster across syllables from its existence in real French words.

A most recent argument comes from Maïonchi-Pino et al. (2020), who used intervocalic C_1_C_2_ clusters with null or quasi-null orthographic and phonological statistical values (e.g., “VG” in “UVGOZE”) in a short-term developmental study. Maïonchi-Pino et al. (2020) designed their stimuli following the acute Gouskova’s (2004) proposal of a gradient-based formalization of the Syllable Contact Law—and hence the Sonority Sequencing Principle—in terms of *phonological sonority-based markedness constraints* implemented within the Optimality Theory (e.g., Prince and Smolensky, 2004). Within the Optimality Theory, Gouskova’s (2004) syllable contact is a stratified relational hierarchy that determines the well-formedness of a coda or onset not in isolation but in relation to the adjacent individual sonorities of onset and coda, respectively. Therefore, an SP is not sensitive to the type of consonant (i.e., fricative, nasal, obstruent, etc.) but to the sonority distance (i.e., the sonority rise or fall of from C_1_ to C_2_), so /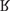
d/and/mt/belong to the same stratum and are theoretically equivalent, as these clusters share the same sonority distance (i.e., *s* = −4; see Supplementary Figure 2). A syllable contact preferentially exhibits a steep fall in SP across syllable boundaries (unmarked, most well-formed; e.g., high-fall SP such as /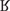
b/, *s* = −4); syllable contacts progressively become marked, and ill-formed as the SP increases across syllable boundaries from high-fall SP to low-fall SP (e.g., /df/, *s* = −1), then on to plateau SP (i.e., null distance; e.g., /bd/, *s* = 0), low-rise SP (e.g., /ml/, *s* = +1), and high-rise SP (e.g., /b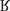/, *s* = +4; i.e., /lb/ > /bd/ > /bl/; >stands for “preferred over”). The markedness pattern across syllable boundaries is, therefore, the complete opposite of the markedness pattern in onset clusters. Outstandingly, the results of Maïonchi-Pino et al. (2020) perfectly echo to Gouskova’s (2004) syllable contact: beginning readers from first grade increasingly—and automatically—conformed to optimal syllable boundaries with intervocalic C_1_C_2_ clusters following a continuum from illegal, marked SPs in which children promoted optimal syllable onsets (i.e., “high-rise” SPs; e.g., “DM” as “U.DMUBE”) to legal, unmarked SPs (i.e., “high-fall” SPs; e.g., “VG” as “UV.GOZE”).

The present study tries to further confirm whether the location and segmentation of intervocalic C_1_C_2_ clusters in skilled adult readers who benefited from massive experiences with phonological and orthographic co-occurrences depend on universal phonological grammar. To do so, we used an *illusory conjunction* paradigm (IC, henceforth) with C_1_C_2_ clusters that mostly do not exist or are not allowed in onset position, as they are phonotactically illegal in French; see Dell (1995)—from marked SPs to unmarked SPs within written pseudowords to *bypass*—or at least to suppress—the language-specific salience of letter and sound co-occurrences. Although Maïonchi-Pino et al. (2020) only *a posteriori* analyzed the potential influence of acoustic–phonetic parameters (e.g., voicing, place- and manner-of-articulation of the intervocalic clusters, etc.), the present study tried to neutralize upstream—or at least disrupt—the potential activation of acoustic–phonetic properties on the expression and the amplitude of the (mis)perception of C_1_C_2_ clusters by adding and playing white noise. Playing a white noise is expected to interfere with the acoustic–phonetic representations if sonority is a functionally grounded linguistic constraint derived from speakers’ linguistic experience. We used two versions of the IC paradigm (e.g., Prinzmetal et al., 1986, 1991). This allows investigating whether syllables are units quickly and automatically activated from visual perception. An IC is a misperception of the color of a target-letter. ICs are supposed to reflect which sublexical structure of letter strings emerge at the perceptual level of (pseudo)word processing. Participants are required to report the color of a target-letter embedded in a bicolored (pseudo)word. In a word like “CARTON” *“BOX”* presented either as “CARTon” or “CARton” (where upper- and lower-case letters represent two different colors), there are: (1) ICs that preserve the syllable boundary (i.e., the participant reports that the target-letter “T” is the same color as “on” in “CAR.Ton”; the dot represents the syllable boundary); and (2) ICs that violate the syllable boundary (i.e., the participant reports that the target-letter “t” is the same color as “CAR” in “CAR.ton”). If syllable units are activated in visual (pseudo)word processing, preservation ICs are expected to exceed violation ICs. Our two main predictions were thus as follows: (1) if the (mis)perception of intervocalic C_1_C_2_ clusters depends on phonological sonority-based markedness sensitivity, we expected that preservation ICs should increase as markedness decreases (i.e., from marked high-rise SPs to unmarked high-fall SPs); (2) if acoustic–phonetic and distributional properties are optional for segmentation, we expected that white noise played during the task and suppressing orthographic and phonological distributional information should not interfere with the expression of phonological sonority-based markedness sensitivity.

## Materials and Methods

### Participants

Sixty-four French native speaker adults participated in Task 1 (*M*_*age*_ = 19.7 ± 1.8; sex ratio: 56 women, 8 men), and 64 French native speaker adults participated in Task 2, but they were different from those who participated in Task 1 (*M*_*age*_ = 19.3 ± 1.2; sex ratio: 57 women, 7 men). All had to complete and sign an informed consent form (no language impairment, no bilingualism, or extensive practice of a second language was reported). All were recruited at Université Clermont Auvergne and received a credit course. All had normal or corrected-to-normal vision and hearing. All were right-handed (+0.80 and +1 right-handedness scores were measured with the Edinburgh Handedness Inventory; Oldfield, 1971). This study received approval from local ethics committees.

### Material

We used the same material as in Maïonchi-Pino et al.(2020; see Appendix; Supplementary Material) and in both Task 1 and Task 2. All the method that describes how the stimuli were created and pretested is fully detailed in the Material section of the Maïonchi-Pino et al. (2020) paper.

Here, we remind that the C_1_C_2_ clusters were divided into 5 SPs (7 pseudowords × 5): high-fall (e.g., /md/; *s* = −4, −3, or −2), low-fall (e.g., /fk/; *s* = −1), plateau (e.g., /kp/; *s* = 0), low-rise (e.g., /zm/; *s* = +1), and high-rise (e.g., /zr/; *s* = +2 or +3). Intervocalic cluster markedness fell from high-rise SPs (the most marked and most ill-formed) to high-fall SPs (the least marked and most well-formed). Each SP comprised seven different C_1_C_2_ clusters.

The experimental conditions were as follows: Two colors (red and blue) were assigned to two different segments of a pseudoword. No two segments ever had the same color. This resulted in two experimental conditions. In the color-syllable compatibility condition, the color segmentation matched the expected syllable segmentation (e.g., “UL.byre”), whereas in the color–syllable incompatibility condition, the color segmentation mismatched the expected syllable segmentation because segmentation occurred either before (e.g., “Ul.byre”) or after (e.g., “UL.Byre”) the syllable boundary. The target-letters to be detected were either the second or the third letter within the syllable boundary and were always at the border of the colored segments to prevent lateral masking (e.g., “L” or “b” in “ULbyre”; “l” in “Ulbyre”; “B” in “ULByre”). Each pseudoword was repeated four times (*N* = 140): twice in the color–syllable compatibility condition (i.e., “L” in “ULbyre” and “b” in “ULbyre”) and twice in the color–syllable incompatibility condition (i.e., “l” in “Ulbyre” and “B” in “ULByre”). In the color–syllable compatibility condition, as color and syllable boundaries matched, only *violation ICs* occurred when the color of “L” or “b” in “ULbyre” was misperceived as being the same color as “byre” or “UL,” respectively. In the color–syllable incompatibility condition, as color and syllable boundaries mismatched, only *preservation ICs* occurred when the color of “l” in “Ulbyre” was misperceived as being the same color as “U” or when the color of “B” in “ULByre” was misperceived as being the same color as “yre.”

### Procedure

Participants were tested in a 15-min session (*M*_*session*_ = 15 min ± 1.7). We used E-Prime 2.0 Professional software (Schneider et al., 2002) to design, compile, and run two scripts on Sony X-series laptop computers under Windows 7 OS. They sat 57 cm roughly from the screen in a silent room. Each trial proceeded as follows: a vertically centered green square (0.82° of visual angle) was displayed for 250 ms and was replaced by a black uppercase target-letter, which was displayed in the center of the screen for 1,250 ms (0.41° of visual angle). This was followed by a 250-ms white screen replaced by a two-colored pseudoword, which covered 2.46° of visual angle, flashed up at a visual angle of 1.14° from the screen. A black question mark (?) then appeared in the center of the screen where it remained until the participant responded (after 5,000 ms, a warning message indicated the absence of response; in Task 1, *N* = 19, 0.21%; in Task 2, *N* = 23, 0.29%; the “no-responses” were excluded from the analyses—and the next trial was started). The next trial followed after a 750-ms delay. In Task 2, white noise was simultaneously played—and adjusted—when the pseudoword flashed.

We applied a two-step adjustment for the duration; first, the participants were trained with a practice list on 18 trials and received corrective feedback (no feedback was given in the experimental lists). The initial exposure duration in the practice list was set to 217 ms (13 refresh cycles of 16.67 ms). The exposure duration was adjusted every three trials in the practice list in decrements and increments of one refresh cycle. Second, the mean exposure duration for each participant, at each time, was used to set the initial exposure duration in the first experimental list (*M*_*duration*_ in Task 1 = 194 ms ± 14; *M*_*duration*_ in Task 2 = 191 ms ± 15). For the experimental lists, the increment/decrement procedure was the same as for the practice list (*M*_*duration*_ in Task 1 = 161 ms ± 19; *M*_*duration*_ in Task 2 = 168 ms ± 21). IC rate was constant ± 20–25% throughout the task.

The pseudowords in the color–syllable compatibility and syllable–color incompatibility conditions were counterbalanced across five experimental lists separated by pauses. The distribution of the pseudowords was pseudo-randomized, whereas the order of their presentation was randomized. To avoid decision bias in the experimental trials, we inserted two fillers after each pause (*N* = 10), i.e., at the beginning of each experimental list, and the corresponding results were not included in the statistical analysis. The participants were instructed to report the color of the target-letter in the flashed pseudowords as quickly and accurately as possible. They had to press on the “blue” or “red” response keys (“r” or “n” buttons, respectively). The software automatically recorded ICs and response times.

## Results

Our 2 × 2 × 5 × 2 mixed design was submitted to JASP 0.13 software (JASP Team, 2020). We entered individual responses (correct = 1, error = 0) for each item into a generalized linear mixed-effects model analysis (128 participants × 140 items = 17,920 data – 42 “no-responses”). We selected a binomial distribution with a Logit link function. The significance of the fixed effects was determined with type III model comparisons. PARTICIPANTS and ITEMS were considered as random factors, whereas CONDITION (compatibility; incompatibility), TARGET-LETTER POSITION (second letter; third letter), SONORITY PROFILE (high-fall; low-fall; plateau; low-rise; high-rise), and CONTEXT (normal; white noise) were entered as fixed factors. The error of approximation decreased by 9.1% (*R^2^_*marg*_* = 0.091), thanks to the fixed effects, whereas all effects together decreased the error of approximation by 13.5% (*R^2^_*cond*_* = 0.135). AIC and BIC were 12,855 and 13,183, respectively, in a successful model convergence (bobyqa optimizer). Before the analysis, we used two-step data standardization. Because we were interested in early, quick responses, we applied a restrictive procedure for data inclusion. First, correct response times ≤ 300 and ≥ 3,000 ms were considered as ICs (0.69% of the data); then, correct response times were trimmed (i.e., for each subject, response times that deviated by ±1.5 standard deviations from the mean were considered as ICs; 0.13% of the data). IC rate represents ± 10.95% of the data. Descriptive data are summarized in Table 1.

**TABLE 1:** Descriptive data for the CONDITION × SONORITY PROFILE × TARGET-LETTER POSITION × CONTEXT.

	**Task 1**				**Task 2**			
	**Color-syllable compatibility**		**Color-syllable incompatibility**		**Color-syllable compatibility**		**Color-syllable incompatibility**	
	**Second letter**	**Third letter**	**Second letter**	**Third letter**	**Second letter**	**Third letter**	**Second letter**	**Third letter**
High-fall	6.4	9.0	18.2	18.6	3.8	8.0	19.6	13.0
Low-fall	4.1	10.5	16.0	14.4	2.5	11.2	16.5	14.1
Plateau	9.2	7.9	15.5	11.8	4.2	16.7	15.2	10.8
Low-rise	15.7	9.8	10.7	15.7	5.2	10.4	11.9	13.2
High-rise	18.6	7.8	10.0	15.3	20.3	2.7	7.2	17.2
*Mean*	10.8	9.0	14.1	15.2	7.2	9.8	14.1	13.7

*Responses are given in percentage (%) for ICs. Errors in the color–syllable compatibility correspond to violation ICs; errors in the color–syllable incompatibility correspond to preservation ICs.*

### Discrimination Sensitivity Threshold and Decision Criterion Analysis

First, to determine the discrimination sensitivity threshold (*d’* value; i.e., this indicates to what extent the participants experienced difficulties to decide the color of the target-letters) and the decision criterion (β value; i.e., this estimates to what extent the [mis]perception of the target-letters within the two-colored pseudowords was biased toward a syllable segmentation), we referred to the *signal detection theory* (e.g., Macmillan and Creelman, 2005). We ran pairwise Student’s *t*-tests on both the *d’* value and β value computed for each CONTEXT (normal vs. white noise). We did not find a significant—but a marginally—difference (*p* > 0.06) for the discrimination sensitivity threshold between normal CONTEXT (*M* = 2.73 ± 0.46) and white noise CONTEXT (*M* = 2.56 ± 0.51); none of the participants had a *d’* = 0 ± 5% (*d’* = 0 ± 5% means *random responses* embedded between 47.5 and 52.5%). The distribution for the *d’* values was scattered around 2.5 (*M* = 2.65 ± 0.49; *min* = 0.34, *max* = 4.05); this indicates that the detection was overall difficult, with extreme variations from low to high sensitivity thresholds. However, we observed a bias toward syllable segmentation only for the distribution for the β criterion in normal CONTEXT [*M* = 1.01 ± 0.72; white noise CONTEXT, *M* = 1.94 ± 1.01; significant difference: *t*(126) = 5.98, *p* < 0.0001]. Overall, values for the *d’* were ranked from very low sensitivity with a very difficult detection (*min d’* = 0.34) to high sensitivity with easy detection (*max d’* = 4.05), whereas values for the β were ranked from high liberalism (*min* β = 0.19) to high conservatism (*max*β = 7.25).

### Illusory Conjunctions Analysis^2^ ^,3^

**FIGURE 1:**
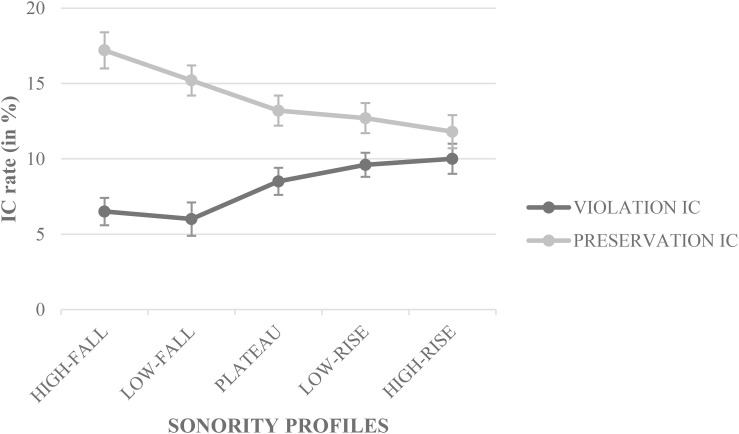
Illusory conjunction rate (IC) in percentage (%) for the CONDITION × SONORITY PROFILE interaction.

Results showed a significant main effect of CONDITION; whatever the context—the CONDITION × CONTEXT did not interact, *p* > 0.05—preservation IC rate (*M* = 13.9% ± 0.61) was higher (Δ = 5.9%, *z* = 12.10, *p* < 0.0001, 95% CI [0.848, 0.928], odds ratio = 1.867) than violation IC rate (*M* = 8.0% ± 0.43). The CONDITION × SONORITY PROFILE interaction was significant (*z* = 5.58, *p* < 0.0001, 95% CI [0.803, 0.947], odds ratio = 0.400; Figure 1); a *post hoc* test (with *p*Bonferroni’s adjusted α level for significance) revealed that preservation ICs decreased continuously from high-fall SPs (*M* = 17.2% ± 1.23, 95% CI [0.803, 0.851]) to high-rise SPs (*M* = 11.7% ± 0.99, 95% CI [0.861, 0.900]), whereas violation ICs increased regularly from high-fall SPs (*M* = 6.5% ± 0.69, 95% CI [0.920, 0.947]) to high-rise SPs (*M* = 10.0% ± 0.99, 95% CI [0.878, 0.917]). Most of the differences were significant with the noticeable exception of preservation and violation ICs for low- and high-rise SPs.

**FIGURE 2:**
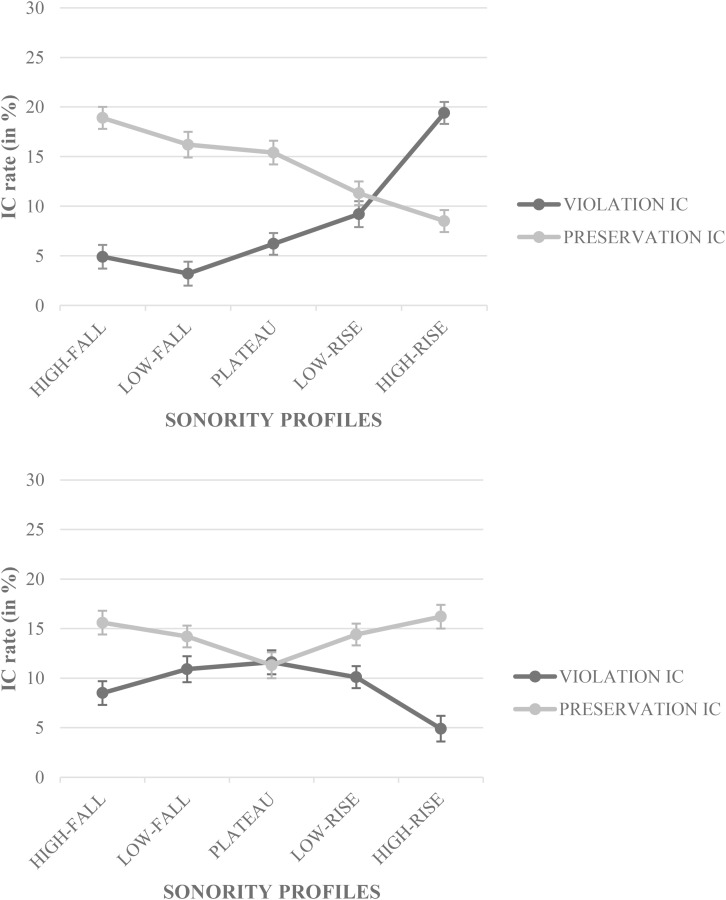
Illusory conjunction rate (IC) in percentage (%) for the CONDITION × SONORITY PROFILE × TARGET-LETTER POSITION interaction as a function of the TARGET-LETTER POSITION (upper panel, the second letter; lower panel, the third letter).

The CONDITION × SONORITY PROFILE × TARGET-LETTER POSITION interaction was significant (*z* = 9.42, *p* < 0.0001, 95% CI [0.772, 0.978], odds ratio = 21.974; Figure 2); a *post hoc* test (with *p*Bonferroni’s adjusted α level for significance) indicated that the IC patterns in the CONDITION × SONORITY PROFILE interaction depended on the TARGET-LETTER POSITION; with a target-letter in the second position (e.g., “L” in “uLBYRE” or “L” in “ULbyre”), preservation ICs decreased from high-fall SPs to high-rise SPs, whereas violation ICs increased from high-fall SPs to high-rise SPs with maximum significant differences between the extreme SPs (i.e., high- and low-fall SPs vs. high- and low-rise SPs). Preservation IC rate significantly outperformed violation IC rates for high-fall, low-fall, and plateau SPs (e.g., “L” in “uLBYRE” was misperceived as “ulBYRE” more often than “l” in “ulBYRE” was misperceived as “uLBYRE”), whereas violation IC rate significantly outperformed preservation IC rate for high-rise SPs only (e.g., “V” in “IVlyde” was misperceived as “Ivlyde” more often than “v” in “Ivlyde” was misperceived as “IVlyde”). With a target-letter in the third position (e.g., “B” in “ULByre” or “B” in “ulBYRE”), preservation ICs followed a V-curve decreasing from high-fall SPs to plateau SPs then increasing to high-rise SPs, whereas violation ICs shape a U-curve, increasing from high-fall SPs to plateau SPs, then decreasing to high-rise SPs. Preservation IC rate significantly outperformed violation IC rates for high-fall and high-rise SPs (e.g., “B” in “ULByre” was misperceived as “ULbyre” more often than “B” in “ulBYRE” was misperceived as “ulbYRE,” whereas “L” in “IVLyde” was misperceived as “IVlyde” more often than “l” in “IVlyde” was misperceived as “IVLyde”). Because the number of letters in the right or left colored segment was not systematically equivalent (e.g., “u.LBYRE” vs. “ul.BYRE” vs. “ulb.YRE”), we explored the non-significant CONDITION × TARGET-LETTER POSITION interaction (*z* = 1.65, *p* > 0.1, 95% CI [0.843, 0.937], odds ratio = 0.844) in which a *post hoc* test (with *p*Bonferroni’s adjusted α level for significance) showed that preservation ICs did not vary with the target-letter position (i.e., “L” in “u.LBYRE” (target-letter in second position, maximized letters in the right segment, *M* = 13.6% ± 0.71, 95% CI [0.850, 0.877]) vs. “B” in “ULB.yre” (target-letter in third position, maximized letters in the left segment, *M* = 14.3% ± 0.72, 95% CI [0.843, 0.871]).

### *Post hoc* Tests

We *a posteriori* tested the robustness of phonological sonority-related markedness effects and how C_1_C_2_ clusters could have been influenced by acoustic-phonetic properties (i.e., global sensitivity to SPs and syllable segmentation within the C_1_C_2_ clusters^6^). We adhered to the procedure used in Maïonchi-Pino et al. (2020, for more details). The Tukey’s HSD *post hoc* tests that we carried out did not reach statistical significance thresholds regarding distinctiveness due to manner-of-articulation parameters (i.e., voicing, *p* > 0.08; sonorance, *p*_*s*_ > 0.09; continuancy, *p*_*s*_ > 0.1; stridency, *p* > 0.1; nasality, *p* > 0.1; mode [dis]similarity, *p* > 0.1) or to place-of-articulation (*p* > 0.08), letter confusability (*p* > 0.1; DRC Letter Confusability Calculator^7^), sublexical frequency distance (*p* > 0.1), and phonotactic transitional probabilities (*p* > 0.1; Crouzet, 2000).

## Discussion

The present study assessed whether phonological universals are credible—complementary —alternatives to an all-statistically based approach to account for the syllable-based effects in French skilled adult readers. Using two versions of the illusory conjunction paradigm (i.e., IC) with and without additional white noise, we gauged how universal phonological sonority-based markedness constraints guide the processing of intervocalic C_1_C_2_ clusters across syllable boundaries with null or quasi-null statistical—distributional—information. Whatever the version of the IC paradigm, we found robust syllable-based effects basically driven by the well-formedness of the C_1_C_2_ cluster sonority profiles.

Our main results show that syllable segmentation does not necessarily depend on statistical—distributional—information. Although our results do not challenge early, implicit knowledge and sensitivity to phonological and orthographic regularities, skilled adult readers do not need language-specific orthographic and phonological statistical information to decide whether intervocalic C_1_C_2_ clusters should be dissociated to form a syllable boundary or should be grouped to form an onset. Indeed, segmentation strategies seem to be guided by the phonological well-formedness of the C_1_C_2_ clusters within the syllable boundaries. As sonority-based markedness progressively increases from high-fall SPs (e.g., “RZ”), i.e., the unmarked and most well-formed intervocalic clusters, to marked high-rise SPs (e.g., “DM”), i.e., the marked and most ill-formed intervocalic clusters, there is a gradual switch from preservation ICs to violation ICs. This progressive crossover actually points out that such a sonority-based segmentation for intervocalic clusters depends on the consonant to be detected (i.e., C_1_ or C_2_). That violation ICs with the second letter dramatically increased with low- and high-rise SPs, whereas preservation ICs decreased, indicates abilities to prefer sonority-based well-formedness of onset clusters over sonority-based ill-formedness of syllable boundaries (e.g., “OGmuze” misperceived as “Ogmuze” more than “Ogmuze” misperceived as “OGmuze”). This is likely true because this does not transgress language-specific phonotactic constraints that rule French (i.e., onset clusters are tolerated as long as they do not exceed a certain number of consonants, i.e., *Maximum Onset Principle*; e.g., Kahn, 1976). However, the fact that violation ICs with the third letter decreased with low- and high-rise SPs, whereas preservation ICs increased, is not in contradiction with response patterns found with the second letter. In addition, there is no robust *cue to consider acoustic–phonetic bias here to account for a random-like* (mis)perception. Rather, these response patterns concur with strict avoidance of complex codas and sonority-based ill-formedness of coda clusters even if this leads to promote simple coda-onset sequences that disrespect, for instance, the Sonority Sequencing Principle (Clements, 1990; e.g., “OGMuze” misperceived as “OGmuze”). Of interest is that, *in fine*, this illustrates the threefold integration of the Syllable Contact Law, the Sonority Sequencing Principle, and the Maximum Onset Principle with a sonority-based well-formedness preference of intervocalic clusters for syllable boundaries (VC_1_.C_2_) and onset clusters (V.C_1_C_2_) over coda clusters (VC_1_C_2_). This preference could lie in French phonotactics in which VC_1_C_2_ syllables (1.9%) are extremely rare compared with C_1_C_2_V syllables (14%; Léon, 2007); this is also coherent with developmental data showing that coda clusters are acquired later and after onset clusters (e.g., Demuth and McCullough, 2009). Although there was no orthographic and phonological language-specific information available, the decision to segment or associate C_1_C_2_ is supposed to reflect optimized abstract phonological representations (e.g., Berent, 2013).

These response patterns are also clear arguments that both universal and language-specific constraints co-contribute to syllable-based effects, even without a statistical and distributional cue. However, we do not learn about the time course of universal phonological sonority-based markedness constraints. We still ignore when—and how—both sources of information compete or prevail over other activations.

What could be an *ad hoc* interpretation to be further tested derives from analogies with how the Optimality Theory maps the input to the output (Prince and Smolensky, 2004). We hypothesize that universal and language-specific phonological constraints are co-activated and interact along with the orthographic representations. Then, skilled readers would face two abstract-universal confrontational forces, which drive languages to prefer unmarked structures (i.e., markedness constraints underlain by sonority-related properties) and incite languages to match as close as possible phonological representations to their orthographic representations (equivalent to the faithfulness constraints). Unattested clusters have no straightforward phonological representations in a target language, but the phonological system does not fail to locate and segment C_1_C_2_ clusters. Indeed, the misperception (i.e., IC response patterns) would stem from an active process that phonologically decodes and recodes them into well-formed ones. This is possible after successfully passing through the successive language-specific hierarchically ranked constraints of the universal set of constraints. Hence, the decision to segment or group C_1_C_2_ clusters would aim to faithfully select an abstract phonological sonority-based representation that minimally transgresses—or maximally respects the constraints. However, we concede that skilled adult readers who experience long exposures to oral and written representations could have derived undefined, unspecified abstract phonological or statistical features from the French language (e.g., Albright, 2009; i.e., *Sonority Projection*; e.g., Hayes and Wilson, 2008; Daland et al., 2011). Therefore, this would conform to the assumption that all the sonority-based representations are not fully available but could be implicitly learned and generalized from experiences with phonotactic constraints in language-specific exemplars (e.g., Hayes, 2011).

To date, that sonority lies in speakers’ linguistic experience of the acoustic–phonetic properties of sounds remains unclear (e.g., Hayes and Steriade, 2004; Parker, 2017) as well as the acoustic–phonetic transformations that speech undergoes (e.g., Kirchner, 2004; Davidson, 2011; Davidson and Shaw, 2012). Also, this is far less clear how—and why—acoustic–phonetic properties would mediate reading processes (e.g., Berent and Lennertz, 2010; Tamási and Berent, 2015). However, like Maïonchi-Pino et al. (2020), we did not find *a posteriori* effects of acoustic–phonetic features (i.e., manner- and place-of-articulation), so the IC patterns do not result from low-level similarities in gestural, spectral, or acoustic–phonetic contrasts (e.g., Stevens, 2002). When Maïonchi-Pino et al. (2020) speculated that IC patterns could not stem from spectral or acoustic–phonetic failures to encode and decode the C_1_C_2_ clusters, we indeed showed that white noise did not interfere with the sonority-based IC patterns, the pseudoword display duration, or the IC rates, which were identical to those found with the no-white noise IC paradigm. Here again, we doubt whether sonority-based IC response patterns are modulated by a gestural failure to articulate the C_1_C_2_ clusters, as there is no reliable evidence that skilled adult readers articulate, need to articulate, or have time to articulate them (e.g., Wright, 2004; Redford, 2008).

## Conclusion

Taken together, our results emphasize the importance of phonological universals for segmenting—or associating—intervocalic C_1_C_2_ clusters in visual (pseudo)word processing. For the first time, we found evidence of response patterns to sonority-based markedness constraints in skilled adult readers in French. We replicated those found in beginning readers (e.g., Maïonchi-Pino et al., 2020) and extended those previously released in speech perception either in adults or children, with or without developmental dyslexia (e.g., in English, Russian, and Korean, Berent et al., 2007, 2008, 2011, 2012a; Ettlinger et al., 2012; Hunter, 2019; in Spanish, Berent et al., 2012b; in French, Maïonchi-Pino et al., 2013, 2015b; in Mandarin Chinese, Zhao and Berent, 2016; in Hebrew, Berent et al., 2012c, 2013).

Although syllable-based effects in French seem to be guided by an abstract universal sonority-based well-formedness of the C_1_C_2_ clusters across the syllable boundaries, whatever the experience with written and oral language, we confirmed that these effects do not require available statistical—distributional—information and are dissociated from acoustic–phonetic properties (also see in developmental dyslexia Maïonchi-Pino et al., 2013; Berent et al., 2016). We do not dismiss the contribution of statistical—distributional—information; even so, the IC paradigm does not encourage the statistical learning of our C_1_C_2_ clusters, and rather, we draw attention to suggest that segmentation strategies do not necessarily occur as a by-product of statistical properties. Early, automatic phonological activation in skilled readers is unquestionable, but this raises the question of the time course in activating the phonological universal constraints and the language-specific properties. Further research should be interested in understanding how both of them interact, either in children or in adults, to update models of reading (for instance, see the Interactive Activation Model with Syllables—IAS model—which includes an intermediate syllable level that modulates, i.e., inhibits or facilitates, visual word processing; Mathey et al., 2006).

## Data Availability Statement

The raw data supporting the conclusions of this article will be made available by the authors, without undue reservation.

## Ethics Statement

The studies involving human participants were reviewed and approved by Comité de Protection des Personnes. The participants provided their written informed consent to participate in this study.

## Author Contributions

MT, MB, and NM-P designed the study. MT collected the data. MT and NM-P analyzed the data and wrote the first draft of the manuscript. OL and LF equally contributed to read, comment, and approve the submitted manuscript. All the authors contributed to the article and approved the submitted version.

## Conflict of Interest

The authors declare that the research was conducted in the absence of any commercial or financial relationships that could be construed as a potential conflict of interest.

## Footnotes

1 For a complementary description of other sonority-based linguistic principles, see Maïonchi-Pino et al. (2020).

2 Our data set was coded into 1 = correct response, 0 = error (i.e., IC). Confidence intervals (CIs, henceforth, provided into square brackets) refer to correct responses. As we were interested in the ICs, we reported the IC rate using a conversion as follows: (1 - *M*_*correct response*_) ^∗^ 100.

3 Following the suggestion of one of the Reviewers, for illustrative purposes, individual response times for each item were entered into a generalized linear mixed-effects model analysis with the same fixed and random factors. Our results confirmed that the CONTEXT did not influence the response patterns (*p*_*s*_ > 0.1), but we found significant effects for: (1) CONDITION (*z* = 16.34, *p* < 0.0001, 95% CI [839, 1,088], odds ratio = 2.773)—participants were faster in the color–syllable compatibility (*M* = 878 ms) than in the color–syllable incompatibility (*M* = 1,013 ms); (2) CONDITION × SONORITY PROFILE (*z* = 6.87, *p* < 0.0001, 95% CI [794, 1,125], odds ratio = 0.892)—in the color–syllable compatibility, participants were faster with high-fall SPs (*M* = 817 ms ± 72, 95% CI [794, 901]) than with high-rise SPs (*M* = 916 ± 57, 95% CI [903, 991]), whereas in the color–syllable incompatibility, participants were faster with high-rise SPs (*M* = 886 ms ± 93, 95% CI [862, 1,001]) than with high-fall SPs (*M* = 1,034 ms ± 82, 95% CI [992, 1,125]); (3) CONDITION × SONORITY PROFILE × TARGET-LETTER POSITION (*z* = 11.24, *p* < 0.0001, 95% CI [759, 1,188], odds ratio = 13.653).

6 All the *p*-values refer to the ICs that collapsed for both conditions (i.e., color–syllable compatibility and color–syllable incompatibility) because none of the parameters that we tested interacted with CONDITION.

7 http://www.cogsci.mq.edu.au/ssaunder/utils/lc.php.

## Abbreviations

C: consonant
IC: illusory conjunction
SP: sonority profile
V: vowel.
