# Supplementary material for: Sonority as a Phonological Cue in Early Perception of Written Syllables in French
Source: Front Psychol. 2020 Oct 15;11:558443. doi: 10.3389/fpsyg.2020.558443 (PMC7593649; doi:10.3389/fpsyg.2020.558443)
Supplement: Supplementary file 1 [file Data_Sheet_1.docx]

**Supplementary Material for**

**Sonority as a phonological cue in early perception of written syllables in French**

Méghane Tossonian^1^, Ludovic Ferrand^1^, Ophélie Lucas^1^, Mickaël Berthon^1^, and Norbert Maïonchi-Pino^1^

^1^ Laboratoire de Psychologie Sociale et Cognitive (LAPSCO), CNRS UMR 6024, Université Clermont Auvergne, Clermont-Ferrand, France.

**Correspondence**

Norbert Maïonchi-Pino, PhD, Associate Professor

norbert.maionchi_pino@uca.fr

Supplementary material includes Figure 1, Figure 2, and Appendix

Figure 1. Sonority scale (adapted from Jespersen, 1904; p. 186; also see Gouskova, 2004)^[[1]](#footnote-1)^.

Figure 2. Stratified relational hierarchy of coda and onset proposed within the Syllable Contact constraint, where ‘r’ stands for rhotics, ‘t’ for voiceless occlusives, ‘d’ for voiced occlusives, ‘z’ for voiced fricatives, ‘s’ for voiceless fricatives, ‘n’ for nasals, ‘l’ for laterals, and ‘w’ for glides (adapted from Gouskova, 2004).

Appendix. Stimuli used in the experiment as a function of their Sonority profile.

1. The use of 1.5 and 4.5 is based on a suggestion by Parker (2002), although the relative distance between 1 and 1.5 and 4 and 4.5 may not be equivalent. Glides are not represented, in particular since this would not be useful for our study in French. [↑](#footnote-ref-1)
